# Memory-Driven Forensic Analysis of SQL Server: A Buffer Pool and Page Inspection Approach

**DOI:** 10.3390/s25113512

**Published:** 2025-06-02

**Authors:** Jiho Shin

**Affiliations:** Police Science Institute, Korean National Police University, Asan 31539, Republic of Korea; suchme@police.go.kr

**Keywords:** database forensics, transaction log, cache, buffer pool, SQL server, deleted data recovery, sensor-based systems

## Abstract

This study proposes a memory-based forensic procedure for real-time recovery of deleted data in Microsoft SQL Server environments. This approach is particularly relevant for sensor-driven and embedded systems—such as those used in IoT gateways and edge computing platforms—where lightweight SQL engines store critical operational and measurement data locally and are vulnerable to insider manipulation. Traditional approaches to deleted data recovery have primarily relied on transaction log analysis or static methods involving the examination of physical files such as .mdf and .ldf after taking the database offline. However, these methods face critical limitations in real-time applicability and may miss volatile data that temporarily resides in memory. To address these challenges, this study introduces a methodology that captures key deletion event information through transaction log analysis immediately after data deletion and directly inspects memory-resident pages loaded in the server’s Buffer Pool. By analyzing page structures in the Buffer Pool and cross-referencing them with log data, we establish a memory-driven forensic framework that enables both the recovery and verification of deleted records. In the experimental validation, records were deleted in a live SQL Server environment, and a combination of transaction log analysis and in-memory page inspection allowed for partial or full recovery of the deleted data. This demonstrates the feasibility of real-time forensic analysis without interrupting the operational database. The findings of this research provide a foundational methodology for enhancing the speed and accuracy of digital forensics in time-sensitive scenarios, such as insider threats or cyber intrusion incidents, by enabling prompt and precise recovery of deleted data directly from memory. These capabilities are especially critical in IoT environments, where real-time deletion recovery supports sensor data integrity, forensic traceability, and uninterrupted system resilience.

## 1. Introduction

In the contemporary digital ecosystem, databases constitute the core infrastructure for storing and operating mission-critical information within both corporate and governmental domains. The Microsoft SQL Server enjoys widespread adoption across diverse industry sectors [1]. From a digital forensic standpoint, these databases simultaneously represent primary evidence sources and analytical targets whenever security incidents or criminal activities occur. Recovering deleted data is, therefore, one of the principal challenges at the outset of any forensic database investigation [2,3]. The timing, scope, and perpetrator of a deletion event often determine the investigative trajectory. Yet, even when the fact of deletion is indisputable, technically demonstrating the exact content of the deleted records—and the feasibility of their restoration—remains a demanding task.

Previous forensic studies on SQL Servers have predominantly adopted static, post-mortem analyses performed after the server is shut down, focusing on disk-based artifacts such as .mdf or .bak files. Representative work includes (i) comparing the residual presence of rows according to deletion commands—e.g., DELETE versus TRUNCATE [3]—and (ii) evaluating recovery success rates under logical versus physical acquisition strategies [4]. Yet all these approaches examine only the offline state of the database, offering no means to analyze traces that may exist in memory immediately after a deletion event. Consequently, any evidence that resides solely in volatile memory is lost, and deletions that are flushed from memory before being persisted to disk leave no recoverable footprint. Moreover, methods that infer residual data by parsing on-disk page structures [3,4] inherently fail when the deleted records vanish before those pages are written back to disk. To overcome these limitations, the present study focuses on an integrated recovery strategy that correlates transaction log entries with memory-resident pages. Pages cached in the SQL Server Buffer Pool can diverge from their on-disk counterparts because of SQL Server’s deferred-write policy. Immediately after deletion, row slots may already be removed, and offsets will be updated in memory while the corresponding .mdf pages remain unchanged. This discrepancy underscores the necessity of a real-time, memory-centric forensic approach, which our research seeks to establish.

This need becomes especially critical in sensor-driven and IoT-based environments, where lightweight SQL engines—such as SQL Server Express or SQLite—are frequently embedded in edge gateways, industrial controllers, or mobile data aggregation units. These systems locally store streaming sensor measurements, event logs, and operational state data, often in unattended or physically accessible deployments. Given their exposure, they are particularly vulnerable to insider attacks or intentional data deletion. In such cases, the malicious erasure of sensor records may obstruct forensic reconstruction, disrupt situational awareness, and compromise the historical integrity of sensor-based processes. Therefore, memory-resident forensic techniques capable of recovering deleted data in real time—before memory is overwritten or flushed—offer significant value in ensuring forensic traceability and operational resilience in sensor-centric infrastructures.

Accordingly, this study proposes a memory-centric, real-time forensic procedure that identifies pages and rows residing in memory while the SQL Server is online, analyzes them, and reconstructs deleted data. The method leverages the sys.dm_os_buffer_descriptors dynamic management view to pinpoint pages that remain cached in the SQL Server Buffer Pool after a deletion event, allowing direct inspection of residual records. In parallel, the fn_dblog function is employed to parse the transaction log, correlating log-level deletion entries with in-memory artifacts. This dual analysis enhances the likelihood of successful recovery and enables a more precise reconstruction of the deletion scenario.

Key contributions of this research are as follows:

- Integrated log-and-page workflow: We establish a unified procedure that organically combines transaction log analysis with page-level inspection, enabling fine-grained tracking of deletion timestamps and residual row structures—capabilities that previous, isolated techniques could not provide.
- Real-time buffer-pool forensics: By verifying page residency in the Buffer Pool, we demonstrate the feasibility of analyzing memory-resident pages immediately after deletion, thereby validating the potential of true in-memory forensics.
- Empirical validation of structural recovery: Through controlled experiments, we show that the proposed procedure can reconstruct the structural layout—and, in many cases, the contents—of deleted records.

This real-time, integrated approach mitigates the inherent limitations of conventional static forensics and offers a rapid, effective response framework for investigating deletion events in live SQL Server environments.

## 2. Related Works

Within digital forensics, the Microsoft SQL Server is a pivotal database platform operated by numerous organizations worldwide; consequently, the extent to which deleted data can be recovered has become a central investigative concern. Prior work in this domain can be grouped into three principal streams:

1. Transaction-log reconstruction: techniques that leverage the transaction log to restore deleted rows [2,5].
2. Physical-file carving: methods that directly parse database files (e.g., .mdf, .ldf, .bak) to extract records lingering in unallocated space [3,4,6,7,8].
3. Residual-data characterization: studies that classify and compare data persistence across different deletion events—such as DELETE, TRUNCATE, and DROP [4].

Among these streams, transaction log-based studies offer the clear advantage of back-tracking deletion events and reconstructing original records as long as the relevant log entries are still available. The seminal work of Haerder and Reuter laid the theoretical foundation for transaction-oriented recovery [2,5], and subsequent research has adapted this paradigm to the SQL Server, demonstrating partial restoration of deleted data from log contents.

Nevertheless, this approach has notable limitations: if the log has already been archived or purged, or if the database is provided in an isolated state without its transaction log—such as after a remote acquisition—the method becomes inapplicable. Even when logs are present, they may not capture the entire row structure, raising the risk of incomplete reconstruction.

As a countermeasure to these limitations, a static analysis approach that directly examines physical files (such as the .mdf file) has been proposed [3,4,6,8]. Because deleted records may linger in the unallocated or Free Space of the collected database files, researchers have developed methodologies to recover such data through binary-level examination using hex editors [3,8]. However, this method is limited by operational constraints: since it requires the server to be shut down to acquire a disk image, it is unsuitable for real-time response and impractical for application on a live, running database. Furthermore, in environments where checkpoints or database reconfigurations occur frequently, deleted data may reside only in memory before it is committed to disk, meaning that a static analysis approach alone might overlook critical deleted information.

Furthermore, research has also been conducted on data recovery using SQL Server’s cache, with attempts being made to analyze residual traces of deletions left in the cache [9]. In this regard, instead of relying solely on log-centric or static analysis approaches, this study proposes a real-time forensic technique that identifies deleted pages within the Buffer Pool—a key caching component of SQL Server while it is active [10]—and employs this information to reconstruct deleted rows.

Specifically, the proposed method first identifies pages associated with deleted data through transaction log analysis [11]. Next, cache-related dynamic management views (DMVs) such as sys.dm_os_buffer_descriptors are utilized to collect pages that still contain residual deleted records in memory [12]. The structure of these pages is then examined directly and further augmented by supplementary analysis of the transaction log to bolster the recovery process. This approach distinguishes itself from traditional methodologies by providing recovery cues from the cache, even in the absence of transaction logs, and by securing data that remains in memory prior to being written to disk. Additionally, it offers the significant advantage of enabling rapid, on-site investigation and analysis without requiring a server restart or the physical acquisition of disk images.

## 3. Background

### 3.1. SQL Server Forensics

The SQL Server is a widely deployed relational database management system on a global scale, making it a central subject in digital forensic investigations when insider threats or external breaches occur. Deletion events, in particular, offer critical clues during investigations and audits, thereby raising important issues regarding how to forensically examine, recover, and reconstruct such events. In SQL Servers, deleted data may temporarily reside in two key components—the cache and the transaction log. The cache stores query execution information, sessions, and page data in memory during server operations and can be inspected in real time via Dynamic Management Views (DMVs), which are system views that expose the internal server state and performance metrics. Conversely, the transaction log is a file that records all DML (Data Manipulation Language) and DDL (Data Definition Language) operations generated by the database, in which deleted rows may be recorded in the form of LOP_DELETE_ROWS.

Previous research has mainly focused on static analyses based on logs or on-disk files (e.g., .mdf and .ldf files), while approaches that actively utilize residual deletion traces within the cache have been relatively underexplored. This section examines, in turn, the DMV cache, the transaction log, and the physical page structure to explore the SQL Server’s internal mechanisms and analytical potential for recovering deleted data.

### 3.2. Transaction Log

The transaction log is a critical component of the SQL Server that records all data modifications executed by DML operations, as well as all definition tasks performed by DDL, all for the purpose of ensuring data integrity and enabling recovery [13]. Typically paired with the database file (.mdf), the transaction log exists as an .ldf file, and its retention depends on factors such as the log backup interval and the recovery model (e.g., Full, Bulk-logged, Simple). For instance, under the Full recovery model, all changes are maintained for an extended period, thereby increasing the likelihood that deleted data remain recorded in the transaction log [11].

Analyzing the transaction log allows for the identification not only of commands such as DELETE or DROP executed at a particular moment but also of the actual data contained in the removed rows. This is possible because the log records the binary state of data both before and after each change. The SQL Server provides the function fn_dblog to inspect the active log area. When the Operation column shows the value LOP_DELETE_ROWS, it indicates that a row was deleted via a DELETE command [14]. Likewise, when the Context column displays LCX_MARK_AS_GHOST, it signifies that the SQL Server has internally marked the row as a ghost record. A ghost record indicates that a row has been deleted and is flagged as such within the page structure—the basic unit of SQL Server data I/O—even though the row’s previous data remain available in the transaction log [15]. In this way, a recovery approach based on transaction log analysis can identify deletion-related operations and extract the actual data of deleted rows from the RowLog Contents column.

However, recovery techniques based on transaction log analysis are difficult to apply if the log has already been backed up and purged, or in environments such as the SIMPLE recovery model, where logs are not retained for long periods. Additionally, since the RowLog Contents are stored as hexadecimal values [14], further processing is required to interpret them in conjunction with the table schema that defines the column structure.

### 3.3. Buffer Pool

#### 3.3.1. Cache

The SQL Server incorporates several memory-based structures—such as the Plan Cache, Buffer Pool, and Procedure Cache—which are collectively known as the “Cache”. This Cache manages elements, including executed query plans, pages loaded from disk files, and session information. 

The primary types of Cache in the SQL Server are as follows:

- Plan Cache: Stores execution plans for recently executed queries.
- Buffer Pool: Space pages read from disk files (e.g., .mdf, .ldf) in the memory.
- Procedure Cache: Retains the results of executed Stored Procedures.

**Figure 1:**
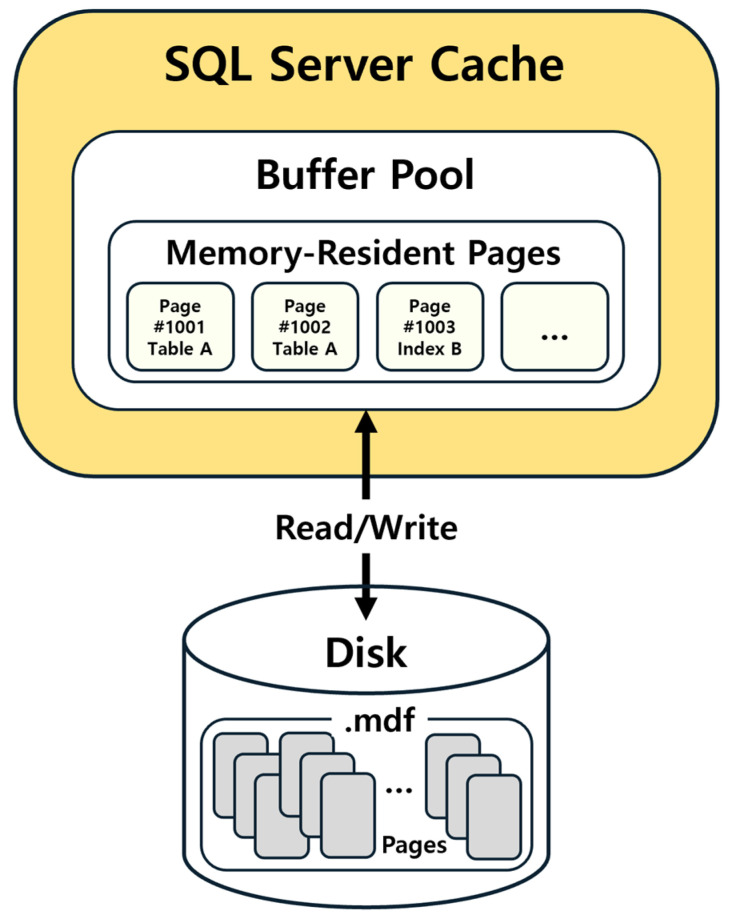
Conceptual diagram of the SQL Server Cache and Buffer Pool.

Caches in the SQL Server are actual memory structures that exist to optimize performance and facilitate rapid access. They can, however, be volatile, as they may be flushed during events such as server restarts or memory pressure. This study focuses specifically on the Buffer Pool. When data are deleted in a particular SQL Server instance, it implies that the corresponding page containing that data remain loaded in the memory’s buffer area [16,17]. Consequently, the aim of this study is to immediately capture and analyze these pages from the Buffer Pool, thereby minimizing data loss and enhancing the potential for recovery. Figure 1 conceptualizes the Buffer Pool within the SQL Server Cache.

#### 3.3.2. DMV

The DMV is the system view interface provided by the SQL Server to query internal structures and cached components [18]. Analysis through DMVs offers substantial advantages from a real-time forensic standpoint. While the SQL Server is actively running, investigators can immediately access session information, query statistics, and lists of memory-resident pages cached in the system—enabling the rapid identification of potentially malicious queries such as DELETE or DROP, along with the user sessions that executed them. Additionally, because DMVs can be accessed using only T-SQL-a Microsoft-specific extension of standard SQL, they offer an efficient way to respond promptly in the aftermath of an incident without requiring any external tools. This memory-based approach stands out from traditional static analysis techniques, as it allows for the capture of volatile traces before they are flushed to disk or removed through log backups.

On the other hand, the volatility of cache structures represents a clear limitation. When the server is restarted, or the cache is flushed, previously executed queries and memory-resident page information may no longer be retrievable via DMVs. In scenarios that require long-term recovery or forensic tracing of deletion events from past time periods, relying solely on cache data becomes impractical. Although DMVs can reveal query execution plans and session metadata, accurately reconstructing the actual contents of deleted rows—the data itself—requires supplementing cache analysis with an examination of either the transaction log or the on-disk pages. Ultimately, while cache analysis offers a powerful means for immediate response and the collection of supplementary evidence immediately after deletion, its limitations in uncovering long-retained evidence must be clearly recognized.

In summary, retrieving information stored in the cache requires querying relevant system views through DMVs using T-SQL. The key DMVs related to cache analysis are summarized in Table 1.

By leveraging DMVs, investigators can obtain real-time information from various perspectives, including deletion queries, memory-resident pages, and user sessions. However, due to the volatility of the cache, timely analysis before a server restart is critical for maximizing effectiveness. When combined with other techniques—such as transaction log analysis or DBCC PAGE—DMVs can enhance the forensic reconstruction of deleted data and strengthen evidentiary support [19]. Among these, this study adopts sys.dm_os_buffer_descriptors as a core component of its recovery methodology.

### 3.4. Page

**Figure 2:**
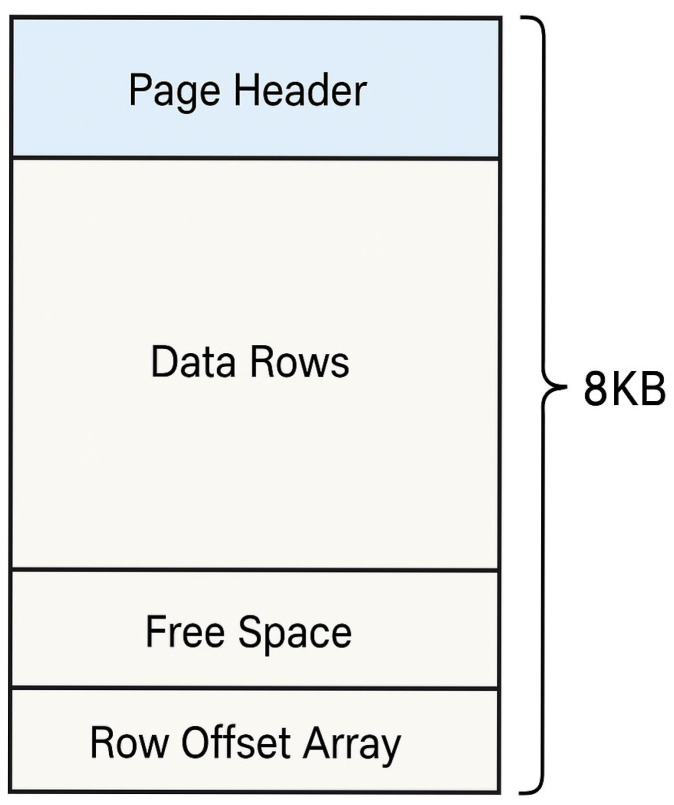
SQL Server data page structure.

SQL Server stores and manages data in 8 KB units known as pages [20]. This page structure is used not only in disk-based database files (e.g., .mdf, .ndf), but also in the memory cache (Buffer Pool) of a running SQL Server instance. As shown in Figure 2, a page is organized with the following structure.

The key components of the page structure are as follows:

- Page Header: Stores metadata such as Page ID, Page Type, and Slot Count.
- Data Rows: Contains the actual record data.
- Free Space: Represents unused space resulting from deletions or updates.
- Row Offset Array (Slot Array): Stores the starting position of each row, with offset values written in reverse order from the end of the page.

The Buffer Pool is the SQL Server’s primary caching area, where frequently accessed pages are loaded into memory. In essence, the term cache in SQL Server typically refers to the collection of pages residing in the Buffer Pool. This mechanism allows SQL Server to access data rapidly without having to retrieve it from a physical disk, thereby significantly improving performance. 

### 3.5. Integrating Transaction Log, Buffer Pool and Page Analysis for Recovery

When a DELETE operation is executed in SQL Server, the deletion event (recorded as LOP_DELETE_ROWS) is first logged in the transaction log file (.ldf) based on its LSN (Log Sequence Number). The page where the deleted data originally resided then undergoes an update to its Row Offset Array. Although the exact behavior may vary slightly depending on the SQL Server version, the offset for the deleted row is typically updated to 0x0000 or replaced with the offset of the next row. The binary content of the actual row may still remain physically in the Data Rows area of the page, and as long as it is not fully overwritten, recovery remains possible.

## 4. Proposed Recovery Methodology

### 4.1. Framework of Proposed Methodology

**Figure 3:**
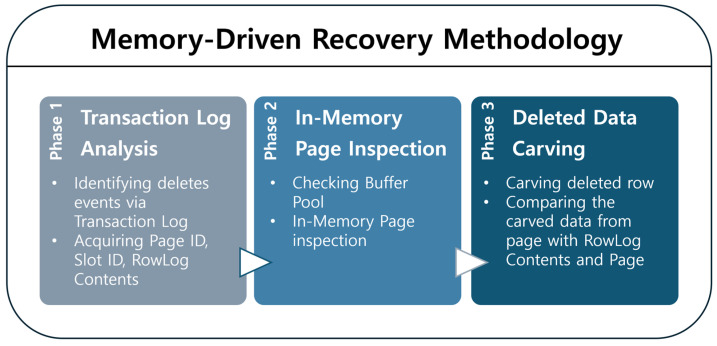
Proposed recovery methodology.

This section presents a practical procedure for recovering deleted data in an SQL Server environment. The proposed methodology combines transaction log analysis (logical trace) with page structure inspection (physical inspection), enabling both the reconstruction of deletion events and the recovery of deleted data by correlating logical deletion history with residual physical records. In particular, the analysis focuses on pages residing in SQL Server’s Buffer Pool, demonstrating the feasibility and applicability of a memory-driven forensic approach. As shown in Figure 3, the proposed recovery procedure consists of three sequential phases.

The deleted row recovery methodology proposed in this study begins by analyzing the transaction log to trace the history of the deletion event and identify metadata such as the Page ID and Slot ID of the deleted row. Next, it verifies whether the corresponding page is currently loaded in SQL Server’s Buffer Pool. If the page is resident in memory, the DBCC PAGE command is used to inspect its structure, allowing investigators to examine the state of the deleted row’s offset and to locate any residual data within the page. In the final phase, the RowLog Contents extracted from the transaction log is compared—at the byte level—with the data remaining on the page to reconstruct the deleted row. This procedure centers on memory-based analysis while leveraging transaction logs to provide contextual information about the deletion event. As a result, it enables a unified interpretation and reconstruction of both the temporal and physical aspects of deleted data. The detailed analysis methods for each phase are described in the following section.

### 4.2. Analysis Method

#### 4.2.1. Phase 1: Transaction Log Analysis

The recovery of deleted data begins with identifying the logical trace of the deletion event. The SQL Server generates transaction logs for all DML operations—including INSERT, UPDATE, and DELETE—and specifically records deletions as operations such as LOP_DELETE_ROWS or LOP_MODIFY_ROW. From a data recovery perspective, transaction logs are considered non-volatile; they are not deleted upon server shutdown, making them a valuable asset for reconstructing the history of actions. However, it is important to note that the frequency and retention of transaction log entries depend on the server’s configured recovery model, which may affect their availability for forensic purposes. The SQL statement below is used to filter deletion events from the log and extract key metadata such as the Log Sequence Number (LSN) and Page ID:

SELECT * FROM fn_dblog(NULL, NULL)

     WHERE Operation = ‘LOP_DELETE_ROWS’;

This query returns the following key information:

- Transaction ID: Identifies the transaction to which the deletion belongs.
- Transaction Name/Context: Indicates the type of executed command (e.g., DELETE).
- Page ID: Specifies the page number where the deleted row was originally stored.
- Slot ID: Denotes the offset position of the deleted row within the page.
- RowLog Contents: Contains a partial snapshot of the deleted record’s column values.

This process is critical for identifying the page where the deletion occurred and for obtaining clues about the structure and pattern of the deleted row. It serves as the starting point for subsequent page-level analysis.

#### 4.2.2. Phase 2: In-Memory Page Inspection

After identifying the unique Page ID of the deleted data through transaction log analysis, the next step is to verify whether that page currently resides in the Buffer Pool cache memory. This verification is performed by querying one of SQL Server’s DMVs, sys.dm_os_buffer_descriptors. By filtering based on the database_id, file_id, and page_id corresponding to the target page, investigators can determine whether the page is actively loaded in memory. The following SQL Statement is used to query sys.dm_os_buffer_descriptors:

SELECT * FROM sys.dm_os_buffer_descriptors

     WHERE database_id = DB_ID(‘*DB Name*’)

     AND file_id = *File ID*

     AND page_id = *Page ID*;

Once it is confirmed that the target page resides in the Buffer Pool, the DBCC PAGE command can be used to examine the real-time structure of the page directly from memory without accessing the disk. While it is also possible to analyze the page by isolating the .mdf file if the page is not present in the Buffer Pool, this study is fundamentally based on memory-driven forensics that operate without shutting down the SQL Server. Therefore, analyzing pages loaded into the Buffer Pool serves as the core analytical process within the proposed methodology.

The content and structure of a page residing in the Buffer Pool can be examined using the DBCC PAGE command. DBCC PAGE is a Database Console Command (DBCC) designed for maintenance and diagnostic purposes rather than for executing DML operations. It operates in read-only mode, making it particularly valuable for digital forensic analysis and internal structural inspection without altering any data. DBCC TRACEON(3604) is a trace flag used to direct the output of DBCC commands to the SQL Server Management Studio (SSMS) console. Without enabling this flag, the output of DBCC PAGE is not displayed directly to the user. The following command demonstrates how to execute DBCC PAGE:

DBCC TRACEON(3604);

DBCC PAGE(DB Name, File ID, Page ID, 3)

When a page is requested via a DBCC command, SQL Server will prioritize the version of the page currently residing in the Buffer Pool, if available. As a result, the output of DBCC PAGE typically reflects the real-time state of the in-memory page, which confirms the feasibility of memory-based analysis.

The primary goals of page inspection include the following:

- Verifying the Page Header for metadata.
- Analyzing the Row Offset Array.
- Investigating the Free Space region to detect residual data.
- Determining whether the page is being served from the Buffer Pool.

Through this analysis, it is possible to determine whether the page is still valid and accessible, and whether it contains remnants of deleted data. In addition, by examining the slot structure, investigators can infer the deletion method—whether the slot was removed or overwritten—and estimate the starting offset of the deleted row.

#### 4.2.3. Phase 3: Recovery and Verification of Deleted Data

In the final phase, the actual recovery of the deleted record is attempted based on the information obtained in the previous two steps. Specifically, the process proceeds as follows. First, the RowLog Contents is compared with the binary contents of the page. Special column values (keyword signatures) extracted from the log are converted into a byte-level format and searched within the page area, and fixed patterns such as integers are prioritized for matching. Next, the slot position and row structure are estimated to reconstruct the data. At this stage, by applying SQL Server’s row structure rules—such as column data lengths, null bitmap, fixed-length values, and variable-length values—it becomes possible to carve each column’s data starting from the offset where the deleted row was originally located. Finally, a verification process is performed on the recovered data. The carved data is compared with the values from the transaction log to cross-validate whether the data obtained from the page matches what was recorded in the transaction log. If the two datasets are consistent, the result can be considered valid as legal evidence.

A summary of the recovery procedure described above is presented in Table 2.

## 5. Experiment

### 5.1. Design of the Experiment

The purpose of this chapter is to evaluate the effectiveness of the proposed methodology by experimentally verifying whether deleted data in a SQL Server environment can be successfully recovered. The experiment is specifically designed to examine the possibility of reconstructing data deleted through user operations by combining transaction log analysis with memory-based page structure inspection. Through this approach, the study aims to assess both the practical applicability and the real-time analytical potential of the method in digital forensic investigation environments.

This experiment is structured around a three-phase recovery procedure that combines log-based logical tracing with page-based physical analysis. Its primary goal is to provide empirical answers to the following key questions:

1. To what extent can deleted data be accurately and precisely reconstructed when transaction log analysis is combined with page structure analysis?
2. Can real-time analysis of pages residing in the Buffer Pool immediately after deletion serve as a viable method for forensic evidence collection and incident reconstruction?

To address these questions, the experiment was designed according to the following three-phase workflow:

- Phase 1: Trace the deletion event and identify the corresponding Page ID using fn_dblog.
- Phase 2: Verify the existence of the page in the Buffer Pool via sys.dm_os_buffer_descriptors and analyze its structure using DBCC PAGE.
- Phase 3: Recover and reconstruct the deleted data by examining the Free Space region within the page and cross-referencing it with log data.

This experimental design not only evaluates the practical applicability of the proposed methodology but also serves to validate the real-world potential of memory-based forensics. In particular, the ability to analyze deleted data in real time without shutting down the server offers significant advantages in security environments that require an immediate incident response, insider threat mitigation, and real-time evidence collection. This highlights the methodology as a highly meaningful and viable alternative in such critical contexts.

### 5.2. Experimental Environment

This section provides a detailed explanation of the environment, database structure, and deletion scenario used to conduct the experiment based on the proposed recovery methodology. The experiment was designed to verify the process of recovering deleted data by combining SQL Server’s transaction log with the Buffer Pool. The setup was configured to meet the minimum requirements for practical application in forensic contexts.

#### 5.2.1. Environment Setup

The experiment was conducted in a single-server PC environment using a Microsoft SQL Server instance. The main configuration details are presented in Table 3.

**Figure 4:**
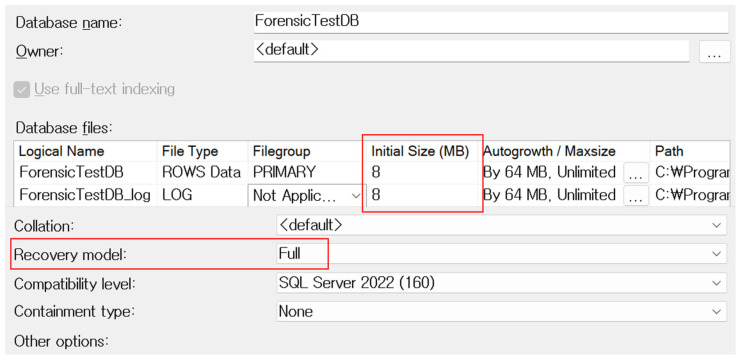
Screen for creating the experimental database instance ForensicTestDB.

To ensure that the real-time state of the Buffer Pool could be analyzed, all experimental procedures were carried out without restarting the server. A database instance named ForensicTestDB was created within the SQL Server catalog. To prevent potential loss of transaction log data due to automatic shrinking or backup operations—and to avoid truncation of unallocated pages—the recovery model was set to FULL. By default, SQL Server configures newly created databases with the FULL recovery model, which provides the most detailed level of logging and recoverability. SQL Server offers three recovery models—SIMPLE, FULL, and BULK_LOGGED—each differing in how transaction logs are retained and how recoverable the data is. The BULK_LOGGED model retains logs for certain bulk operations but may omit detailed row-level changes. In contrast, the FULL model preserves a complete history of transaction logs until an explicit backup is performed, ensuring that all DML operations, including deletions, are thoroughly recorded. This setting is essential for reliably reconstructing deleted rows from transaction logs and for preventing data loss due to automatic log truncation or shrinking processes. Default settings were maintained, including an initial size of 8 MB for both the .mdf and .ldf files, in accordance with the standard SQL Server page size. Figure 4 below shows the settings screen for database creation options.

#### 5.2.2. Table and Data Configuration

A table object named CustomerInfo was created as the target for deletion testing. The table was designed with a simple schema that includes customer identifiers, personal information, and VIP status. The following T-SQL command was used to create the experimental table:

USE ForensicTestDB;

GO

CREATE TABLE CustomerInfo (

CUSTOMER_ID INT IDENTITY(1,1) PRIMARY KEY,

NAME NVARCHAR(100),

PHONE VARCHAR(20),

EMAIL VARCHAR(100),

VIP_STATUS BIT

);

When a large amount of data is inserted, SQL Server may distribute the rows across two or more pages depending on the page structure. To ensure clarity in analyzing the experimental results, the dataset is limited to 10 records, as summarized in Table 4.

#### 5.2.3. Scenario

The experiment was designed under the assumption of an insider threat scenario. In this case, a privileged insider within an organization’s SQL Server environment deliberately deletes sensitive customer information from the system. Upon discovering this activity, a forensic investigator immediately begins examining whether the deleted data have been permanently removed or if remnants still exist within the system. The scenario proceeds in the following sequence:

1. Delete records with VIP_STATUS = 1 using a DELETE statement.
2. Analyze the deletion transaction log using fn_dblog.
3. Verify whether the page containing the deleted data still resides in the Buffer Pool using sys.dm_os_buffer_descriptors.
4. Analyze the page where the deleted row was located using DBCC PAGE, and attempt recovery if the data is still present.
5. Finally, validate whether the recovered data match the data identified through the transaction log.

Dropping a table using the DROP command or initializing it with the TRUNCATE command initializes the Page IDs where the data previously resided. Since the analysis method proposed in this study is centered around tracking specific Page IDs, data deletions performed using DROP or TRUNCATE, which do not allow identification of the relevant Page IDs, are not applicable to the proposed methodology and were therefore excluded from the experiment.

### 5.3. Experimental Results

#### 5.3.1. Transaction Log Analysis

After deleting the records with a VIP_STATUS value of 1, the transaction log was queried using the fn_dblog function, which returned a total of five log entries. The following command was used to perform the query:

SELECT Current LSN,

     Operation,

     Context,

     Transaction ID,

     AllocUnitName,

     Page ID,

     Slot ID,

     RowLog Contents 0

FROM fn_dblog(NULL, NULL)

WHERE Operation = ‘LOP_DELETE_ROWS’

     AND AllocUnitName LIKE ‘%CustomerInfo%’;

In the experimental scenario, the target table was already known in advance, so the AllocUnitName field was filtered by the table name. Additionally, to focus solely on deletion-related events, the Operation field was filtered using the value LOP_DELETE_ROWS. Table 5 presents the transaction log entries retrieved under these conditions.

**Figure 5:**
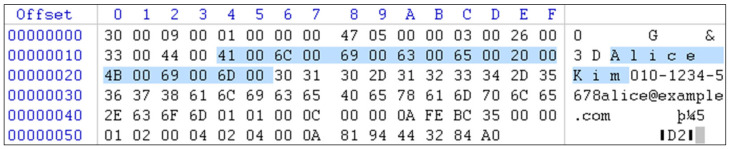
Identifying the row’s signature keyword from the hexadecimal value stored in the RowLog contents field of the transaction log.

The primary objective of transaction log analysis is to identify the specific Page ID where the deleted row was stored. The analysis confirmed that the deleted row resided on Page ID 0x120 with File ID 1. Additionally, it was verified that the row data was stored in hexadecimal format within the RowLog Contents field. As shown in Figure 5, a key string—“Alice Kim”—was extracted from the RowLog Contents as a signature keyword from the deleted row. This serves as a concrete clue that can be used to clearly identify the deleted row and will also aid in subsequent page-level analysis.

#### 5.3.2. Buffer Pool Analysis

**Figure 6:**
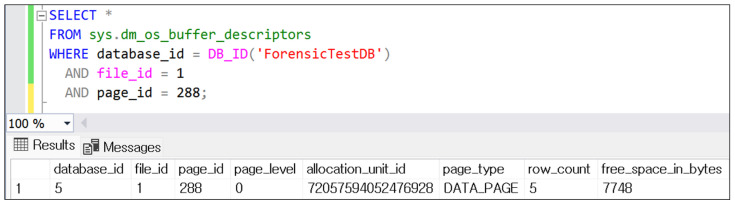
Buffer Pool verification result using DMV (Page ID converted to decimal format).

To verify whether the deleted target page identified through transaction log analysis still resided in SQL Server’s Buffer Pool, the DMV sys.dm_os_buffer_descriptors was queried using the database_id, file_id, and page_id as filter parameters. The query returned a single record, confirming that the corresponding page was indeed present in the Buffer Pool. Figure 6 displays the query result showing the page residing in memory.

#### 5.3.3. In-Memory Page Analysis and Verification of Recovered Data

The structure of the page residing in the Buffer Pool was analyzed using the DBCC PAGE command. The following command was used to extract and inspect the page from the Buffer Pool:

DBCC TRACEON(3604);

GO

DBCC PAGE(‘ForensicTestDB’, 1, 288, 2);

GO

**Figure 7:**
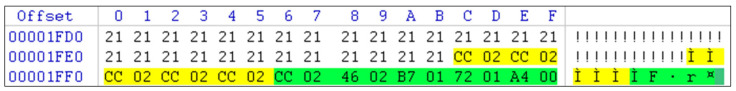
Changes in the Row Offset Array on page 288 after deletion (yellow indicates cleared row offsets; green indicates intact row offsets for undeleted rows).

As a result of analyzing Page 288, it was confirmed that the Row Offset Array values for the five deleted rows had been cleared, as shown in Figure 7. The last row offset had been overwritten with the value 0x2CC, indicating that the deleted entries had been initialized. Additionally, the position of the Slot Array had shifted, further confirming structural changes following the deletion.

However, the deleted row’s data was still present in the Free Space area of the page. This clearly demonstrates that SQL Server’s DELETE operation does not immediately erase the actual row data from the page; instead, it simply resets the corresponding entries in the Row Offset Array. Because the Offset Array is the only part that gets cleared, the deleted row can still be reconstructed by parsing its header and extracting each column value.

**Figure 8:**
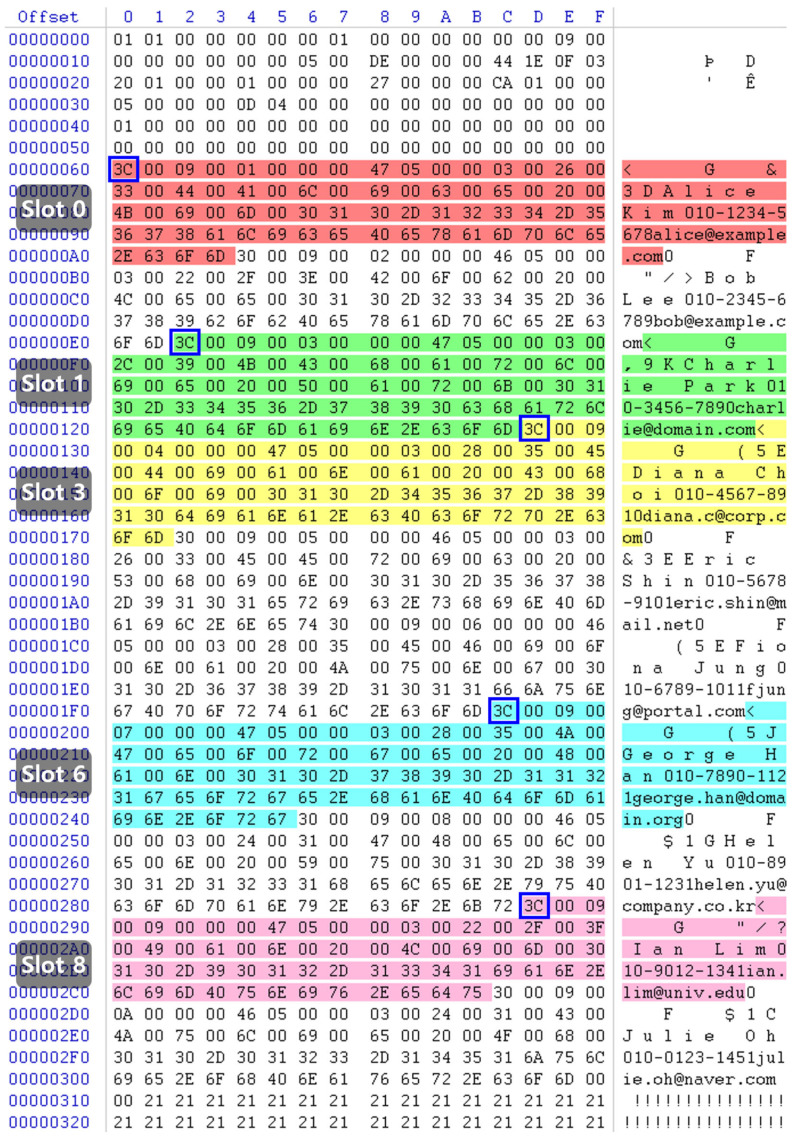
Deleted rows remaining on page 288 (highlighted blocks indicate the deleted rows retained within the page).

A comparison between the RowLog Contents extracted from the transaction log and the residual values of the deleted row within the Buffer Pool page confirmed an exact match. Figure 8 presents the analysis results of the residual state of the deleted rows on Page 288, which precisely aligns with the RowLog Contents previously identified in Table 5. Additionally, the section highlighted in blue in Figure 8 indicates where the row’s STATUS byte was modified as a result of the deletion event. In SQL Server, the row status is stored in the first byte of the row: a value of 0x30 represents a normal (active) row, while 0x3C indicates that the row has been deleted.

### 5.4. Summary

In this study, the recoverability of deleted data was experimentally verified through a combined approach involving transaction log analysis and memory inspection. By analyzing the transaction log, key information such as the deletion event, target Page ID, Slot ID, and RowLog Contents was extracted. When the corresponding page was found to reside in the Buffer Pool, the DBCC PAGE command was used to inspect the Free Space region and the Row Offset structure. As a result, partial or complete remnants of string and numeric data from the deleted rows were observed. In particular, the study demonstrated that structural recovery of deleted records is feasible by comparing the hexadecimal values recorded in RowLog Contents with those found in the Free Space area of memory. This real-time analysis can be performed without requiring a server restart or backup, offering a practical advantage over conventional disk-based or log-only recovery approaches. Table 6 summarizes the distinctive contributions of this study.

As shown in Table 6, this study goes beyond simple log analysis by incorporating real-time memory trace inspection and structural comparison, presenting a recovery approach that ensures both accuracy and timeliness in restoring deleted data. This method provides a practical and distinctive forensic solution compared to traditional approaches, and it is expected to be effectively applicable to insider threat response and incident investigation in future scenarios.

## 6. Limitations

### 6.1. Anti-Forensic Challenges and Their Limitations

It is important to acknowledge that digital forensics is inherently condition-dependent. Its success is determined entirely by the system state and data integrity at the time of acquisition. In extreme cases, anti-forensic activity may completely destroy the object of analysis. For example, in disk forensics, deleted files may be overwritten with null bytes (0x00) or random data, making recovery impossible. Internet history or log files may be replaced with meaningless garbage data, leaving no interpretable artifacts for analysis. In such scenarios, even the most advanced tools may yield no results, and the investigation is halted at the outset.

This reality highlights that digital forensics, despite its technical power, is fragile and context-sensitive. Therefore, forensic methodologies must clearly specify their preconditions and limitations, and research should continue to pursue multi-layered and complementary techniques that utilize memory, logs, metadata, and network traces. Such approaches increase the likelihood of recovery even in partially compromised environments, and reflect a realistic, resilient strategy for modern digital investigations.

A critical limitation of the proposed memory-based recovery methodology lies in its vulnerability to anti-forensic behavior. While the Buffer Pool may temporarily retain deleted records in memory, this assumption can be invalidated in adversarial environments, particularly when the attacker has administrative privileges. In such cases, built-in SQL Server commands may be deliberately used to erase or obscure volatile evidence prior to forensic acquisition.

The SQL Server provides several commands that can purge memory-resident artifacts without generating transaction logs or audit records. For example, DBCC DROPCLEANBUFFERS clears all clean pages from the Buffer Pool, potentially eliminating recently deleted data that has not yet been flushed to disk. Similarly, DBCC FREEPROCCACHE deletes cached execution plans, while DBCC FLUSHPROCINDB removes plan cache entries for a specific database. When executed immediately following a deletion operation, these commands can render both memory-based recovery and query history reconstruction impossible. Table 7 summarizes such commands.

These commands serve as anti-forensic countermeasures, allowing attackers to eliminate critical volatile traces before evidence can be collected. Since they do not leave any transaction-level records, detecting their use post hoc is exceedingly difficult. As such, the practical applicability of the proposed recovery method is contingent upon a defined threat model in which such cache-flushing operations have not occurred. This approach is most suitable in environments where attackers have limited privileges (e.g., insider threats) or where automated forensic response is triggered immediately upon suspicious activity.

### 6.2. Further Research

The proposed recovery technique in the study leverages the fact that deleted data may temporarily reside in the SQL Server Buffer Pool, enabling real-time inspection. However, because the Buffer Pool is a volatile memory structure subject to overwrite due to memory pressure or ongoing queries, there is no guarantee as to how long deleted data will remain intact. In our experiment, recovery was possible for several hours after deletion under a light workload. However, in high-throughput environments, the survivability of deleted data in memory may be significantly reduced. This time sensitivity represents a major limitation of the proposed method. Future research should empirically evaluate the recovery window under various load conditions to strengthen the method’s practical applicability.

Additionally, this study assumes that the memory cache has not been explicitly flushed after deletion. For instance, if a malicious actor issues a DBCC DROPCLEANBUFFERS command immediately following the DELETE operation, the target pages will be removed from the Buffer Pool, rendering the proposed method inapplicable. This highlights that the technique is only effective if the page still resides in memory at the time of forensic analysis. Consequently, this method is particularly well-suited for environments involving insider threats, where the attacker lacks full administrative privileges, or for automated, real-time response systems that capture volatile data before it is lost. Future work should also clearly define the underlying threat model and examine complementary recovery strategies (e.g., log or disk-based methods) when memory artifacts are no longer available.

Finally, the experiment was conducted under relatively controlled conditions and does not reflect the complexity of real-world environments, such as concurrent deletions, page splits, transactional conflicts, or multi-user concurrency. The impact of system events—such as checkpoints, crash recovery, or page eviction—on memory-resident data was also not explored. Furthermore, the dataset used in the experiment was limited in scale compared to enterprise-level deployments. To validate the scalability and general-izability of the proposed approach, future studies should incorporate more comprehen-sive scenarios under dynamic and high-load conditions.

## 7. Conclusions

This study proposed a memory-based forensic analysis procedure for the real-time recovery of deleted data in Microsoft SQL Server environments. Traditional recovery approaches have typically relied on a single method, such as transaction log analysis or disk-based inspection, without offering an integrated framework to compensate for the limitations of each. In response, this study designed a Memory-Driven Recovery Methodology that enables the reconstruction of deleted rows by tracing deletion history through transaction log analysis and directly examining in-memory pages residing in the Buffer Pool.

In particular, the study utilized log-based metadata—such as Page ID, Slot ID, and RowLog Contents—to identify the target page and verify its presence in the Buffer Pool using DMV. The identified in-memory page was then examined using DBCC PAGE, enabling analysis of the Row Offset structure and Free Space region. Based on this inspection, the deleted row data was recovered. Subsequently, the recovered in-page data was cross-referenced with the byte-level information from the transaction log, demonstrating the feasibility of column-level precision recovery. The experiment confirmed that, under the condition that the page remains in memory for a short duration after deletion, cross-analysis of log records and page structures can achieve higher accuracy and real-time responsiveness compared to traditional disk-based methods. Since this recovery procedure can be performed while the database remains online, it significantly improves the practicality and timeliness of digital forensics. In particular, this methodology serves as an effective forensic tool for urgent response scenarios—such as insider deletions or immediate post-breach investigations—enabling fast and highly reliable data recovery. Furthermore, the proposed technique may be particularly beneficial for embedded and sensor-based systems in industrial and IoT environments, where lightweight SQL databases are frequently used to store sensitive measurement data. In such cases, the ability to recover deleted records without interrupting live operations enhances both the forensic readiness and resilience of sensor infrastructures.

Accordingly, the proposed memory-based recovery technique enables real-time restoration of deleted data by leveraging its temporary presence in the Buffer Pool. However, its effectiveness may be limited by various real-world factors, such as the use of anti-forensic commands like DBCC DROPCLEANBUFFERS, memory pressure, concurrent transactions, and system events. Therefore, this method is best suited for environments involving insider threats with limited administrative privileges or automated forensic response systems. Future research should focus on the recoverability of large objects (LOB/BLOB), the development of GUI-based analysis tools, and empirical validation under dynamic and high-load conditions.

## Figures and Tables

**Table 1:** DMV cache views and their advantages and limitations.

Cache View	Description	Advantages	Limitations
sys.dm_exec_query_stats	Retrieving statistical information on previously executed queries	· Measuring the frequency and timing of problematic queries such as DELETE · Identifying recent query execution patterns	· Query text requires additional mapping via sql_handle · Unavailable once evicted from memory
sys.dm_exec_sql_text(sql_handle)	Retrieving the actual query text corresponding to a specific sql_handle	· Identifying specific queries such as DELETE and DROP · Facilitating insider threat tracking	· Highly volatile · Cached queries may become untraceable if replaced in memory
sys.dm_os_buffer_descriptors	List and status of pages loaded in the Buffer Pool	· Determining whether deleted records remain in memory · Real-time verification of Page ID and modification status	· Memory is cleared on server restart or checkpoint events · Detailed row contents require separate page-level analysis
sys.dm_exec_sessions/sys.dm_exec_requests	Information on currently connected users and sessions, including the status of executing queries	· Tracking who is executing DELETE commands in real time · Obtaining connection details such as account name and host name	· Information about past (terminated) sessions disappears quickly · Difficult to preserve snapshots once cache is cleared

**Table 2:** Summary of recovery procedure.

Phase	Procedure	Key Tasks	Tools and Commands	Tarket of Analysis
1	Transaction Log Analysis	· Identifying deletes events · Acquiring Page ID, Slot ID, RowLog Contents	fn_dblog	Transaction Log (LOP_DELETE_ROWS)
2	In-Memory Page Inspection	· Checking Buffer Pool · In-memory page inspection	DMV, DBCC Page	Page Header, Slot Array, Binary Data
3	Deleted Data Carving	· Carving deleted row · Comparing the carved data from page with RowLog Contents	Hex editor	Deleted Row (Value, Offset, Time)

**Table 3:** Experimental environment.

Item	Configuration Details
Operation	Microsoft Windows 11 Pro (64bit), v24H2
RAM/DISK	64 GB/1 TB
SQL Server Version	Microsoft SQL Server 2022 Developer Edition, v16.0.1000.6
Database Name	ForensicTestDB
Recovery Model	FULL
Tool	SQL Server Management Studio (SSMS) v20.2

**Table 4:** Test rows in the experimental table CustomerInfo.

CUSTOMER_ID *	NAME	PHONE	EMAIL	VIP_STATUS
1	Alice Kim	010-1234-5678	alice@example.com	1
2	Bob Lee	010-2345-6789	bob@example.com	0
3	Charlie Park	010-3456-7890	charlie@domain.com	1
4	Diana Choi	010-4567-8910	diana.c@corp.com	1
5	Eric Shin	010-5678-9101	eric.shin@mail.net	0
6	Fiona Jung	010-6789-1011	fjung@portal.com	0
7	George Han	010-7890-1121	george.han@domain.org	1
8	Helen Yu	010-8901-1231	helen.yu@company.co.kr	0
9	Ian Lim	010-9012-1341	ian.lim@univ.edu	1
10	Julie Oh	010-0123-1451	julie.oh@naver.com	0

* The CUSTOMER_ID column was configured as an auto-incrementing field.

**Table 5:** Transaction log query results.

Operation	Transaction ID	AllocUnitName	Page ID	Slot ID	RowLog Contents 0
LOP_DELETE_ROWS	0000:0000040d	dbo.CustomerInfo.PK__Customer__1CE12D377983F610	0001:00000120	0	0x300009000100000047050000030026003300440041006C0069006300650020004B0069006D003031302D313233342D35363738616C696365406578616D706C652E636F6D
LOP_DELETE_ROWS	0000:0000040d	dbo.CustomerInfo.PK__Customer__1CE12D377983F610	0001:00000120	2	0x30000900030000004705000003002C0039004B0043006800610072006C006900650020005000610072006B003031302D333435362D37383930636861726C696540646F6D61696E2E636F6D
LOP_DELETE_ROWS	0000:0000040d	dbo.CustomerInfo.PK__Customer__1CE12D377983F610	0001:00000120	3	0x30000900040000004705000003002800350045004400690061006E0061002000430068006F0069003031302D343536372D383931306469616E612E6340636F72702E636F6D
LOP_DELETE_ROWS	0000:0000040d	dbo.CustomerInfo.PK__Customer__1CE12D377983F610	0001:00000120	6	0x3000090007000000470500000300280035004A00470065006F007200670065002000480061006E003031302D373839302D3131323167656F7267652E68616E40646F6D61696E2E6F7267
LOP_DELETE_ROWS	0000:0000040d	dbo.CustomerInfo.PK__Customer__1CE12D377983F610	0001:00000120	8	0x300009000900000047050000030022002F003F00490061006E0020004C0069006D003031302D393031322D3133343169616E2E6C696D40756E69762E656475

**Table 6:** Comparison of key analysis methods and the recovery approach proposed in this study.

Analysis Method	Log-Based Analysis	Disk-Based Page Analysis	Proposed Method
**Recovery Scope**	Partial columns	Full columns	**Full row structure**
**Slot Status Inspection**	Not possible	Possible	**Possible**
**Deletion Time Tracking**	Possible (based on LSN)	Not Possible	**Possible** (in conjunction with log)
**Real-Time Capability**	Not possible	Low	**Possible** (when residing in Buffer Pool)
**Tool Dependency**	Some commercial tools required	Manual analysis with hex editor, etc.	**Possible using built-in SQL commands**
**Volatility Awareness**	Low	Low	**High** (assuming live memory state)
**Recovery Reliability**	Moderate	Low to moderate	**High**

**Table 7:** T-SQL commands that can be intentionally used as anti-forensic techniques to remove volatile evidence.

Command	Function	Forensic Implication
DBCC DROPCLEANBUFFERS	Removes clean pages from the Buffer Pool	Eliminates residual deleted records that remain in memory
DBCC FREEPROCCACHE	Clears all cached query execution plans	Erases evidence of previously executed SQL commands
DBCC FLUSHPROCINDB	Flushes cached plans for a specific database	Selectively removes query history related to the target database
DBCC FREESESSIONCACHE	Clears session-specific cache metadata	Disrupts the ability to trace user behavior by session

## Data Availability

The original contributions presented in this study are included in the article. Further inquiries can be directed to the corresponding author.

## References

[1] Fowler K. (2008). SQL Server Forensic Analysis.

[2] Haerder T., Reuter A. (1983). Principles of Transaction-Oriented Database Recovery. ACM Comput. Surv..

[3] Shin J. (2017). Comparison of Remaining Data According to Deletion Events on Microsoft SQL Server. J. Korea Inst. Inf. Secur. Cryptol..

[4] Shin J. (2018). Comparing Recoverability of Deleted Data According to Original Source Collection Methods on Microsoft SQL Server. J. Korea Inst. Inf. Secur. Cryptol..

[5] Choi H., Lee S. (2023). Forensic Analysis of SQL Server Transaction Log in Unallocated Area of File System. Forensic Sci. Int. Digit. Investig..

[6] Park S. (2013). A Research for Record Recovery Method in Database. Master’s Thesis.

[7] Ryu G. (2014). A Study for Recovering Records of Microsoft SQL Server’s Database. Master’s Thesis.

[8] Choi H., Lee S., Jeong D. (2021). Forensic Recovery of SQL Server Database: Practical Approach. IEEE Access.

[9] Nissan M.I., Wagner J., Aktar S. (2023). Database Memory Forensics: A Machine-Learning Approach to Reverse-Engineer Query Activity. Forensic Sci. Int. Digit. Investig..

[10] Wagner J., Nissan M.I., Rasin A. (2023). Database Memory Forensics: Identifying Cache Patterns for Log Verification. Forensic Sci. Int. Digit. Investig..

[11] The Transaction Log (SQL Server). https://learn.microsoft.com/en-us/sql/relational-databases/logs/the-transaction-log-sql-server.

[12] sys.dm_os_buffer_descriptors (Transac-SQL). https://learn.microsoft.com/en-us/sql/relational-databases/system-dynamic-management-views/sys-dm-os-buffer-descriptors-transact-sql.

[13] How Reading the Transaction Log in SQL Server. https://learn.microsoft.com/en-us/answers/questions/1132665/how-reading-the-transaction-log-in-sql-server.

[14] Reading the Transaction Log in SQL Server. https://www.sqlshack.com/how-to-read-a-sql-server-transaction-log/.

[15] SQL Server 2022: Introducing Buffer Pool Parallel Scan. https://learn.microsoft.com/ko-kr/shows/data-exposed/sql-server-2022-introducing-buffer-pool-parallel-scan.

[16] SQL Server 2022: Buffer Pool Scans on Large-Memory Computers. https://blog.sqlauthority.com/2023/05/29/sql-server-2022-buffer-pool-scans-on-large-memory-computers/.

[17] Dynamic Management Views and Functions. https://learn.microsoft.com/en-us/sql/relational-databases/system-dynamic-management-views/system-dynamic-management-views.

[18] DBCC (Transact-SQL). https://learn.microsoft.com/en-us/sql/t-sql/database-console-commands/dbcc-transact-sql.

[19] Pages and Extents Architecture Guide. https://learn.microsoft.com/en-us/sql/relational-databases/pages-and-extents-architecture-guide.

[20] Ghost Record Cleanup Process Guide. https://learn.microsoft.com/en-us/sql/relational-databases/ghost-record-cleanup-process-guide.

